# A Randomized Placebo-Controlled Clinical Trial to Evaluate the Medium-Term Effects of Oat Fibers on Human Health: The Beta-Glucan Effects on Lipid Profile, Glycemia and inTestinal Health (BELT) Study

**DOI:** 10.3390/nu12030686

**Published:** 2020-03-03

**Authors:** Arrigo F.G. Cicero, Federica Fogacci, Maddalena Veronesi, Enrico Strocchi, Elisa Grandi, Elisabetta Rizzoli, Andrea Poli, Franca Marangoni, Claudio Borghi

**Affiliations:** 1 Atherosclerosis and Hypertension Research Group, Medical and Surgical Sciences Department, Sant’Orsola-Malpighi University Hospital, Building 2-IV Floor, Via Albertoni 15, 40138 Bologna, Italy; federicafogacci@gmail.com (F.F.); maddalena.veronesi@unibo.it (M.V.); enrico.strocchi@unibo.it (E.S.); elisa.grandi@unibo.it (E.G.); elisabetta.rizzoli@unibo.it (E.R.); claudio.borghi@unibo.it (C.B.); 2 Nutrition Foundation of Italy, Viale Tunisia 38, 20124 Milan, Italy; poli@nutrition-foundation.it (A.P.); marangoni@nutrition-foundation.it (F.M.)

**Keywords:** beta-glucan, fiber, lipid profile, cholesterol, intestinal function

## Abstract

The Beta-glucan Effects on Lipid profile, glycemia and inTestinal health (BELT) Study investigated the effect of 3 g/day oat beta-glucans on plasma lipids, fasting glucose and self-perceived intestinal well-being. The Study was an 8-week, double-blind, placebo-controlled, cross-over randomized clinical trial, enrolling a sample of 83 Italian free-living subjects, adherent to Mediterranean diet, with a moderate hypercholesterolemia and a low cardiovascular risk profile. Beta-glucans reduced mean LDL-Cholesterol (LDL-C) levels from baseline by 12.2% (95%CI: −15.4 to −3.8) after 4 weeks of supplementation and by 15.1% (95%CI: −17.8 to −5.9) after 8 weeks of supplementation (*p* < 0.01 for both comparison and versus placebo). Between baseline and 4 weeks Total Cholesterol (TC) levels showed an average reduction of 6.5% (95%CI: −10.9 to −1.9) in the beta-glucan sequence; while non-HDL-C plasma concentrations decreased by 11.8% (95%CI: −14.6 to −4.5). Moreover, after 8 weeks of beta-glucan supplementation TC was reduced by 8.9% (95%CI: −12.6 to −2.3) and non-HDL-C levels by 12.1% (95%CI: −15.6 to −5.3). Decreses in TC and non HDL-C were significant also versus placebo (respectively *p* < 0.05 and *p* < 0.01 to both follow-up visits). Fasting plasma glucose and self-perceived intestinal well-being were not affected by both beta-glucan and placebo supplementation.

## 1. Introduction

The consumption of dietary supplements to control plasma cholesterol levels has become widespread in recent years, alongside the publication of several systematic reviews and meta-analyses showing the cholesterol lowering effect of some of these products, including fibers [1,2,3].

A comprehensive meta-analysis of 58 clinical trials and 3974 subjects has recently showed that oat beta-glucan significantly affects the serum concentrations of low-density lipoprotein cholesterol (LDL-C), non-high-density lipoprotein cholesterol (non-HDL-C) and apolipoprotein-B (apo-B), concluding that the inclusion of oat-containing foods in the diet may be a valid strategy to prevent the onset of cardiovascular disease [2]. In particular, another meta-analysis presented a dose-response curve between fibre intakes and the reduction of total serum cholesterol, estimating a mean reduction for total cholesterol (TC) and LDL-C levels of 0.045 mmol/L and 0.057 mmol/L respectively, for each gram of dietary fiber [1]. These effects can theoretically play a major preventive role among the general population, since each 1% reduction in TC or LDL-C corresponds to an equivalent 1% decrease in the risk of developing a coronary heart disease event over time [3].

The mechanisms underlying the lipid-lowering properties of dietary fiber are still not fully understood [4]. The ability of soluble dietary fiber to form viscous solutions that prolong gastric emptying, and inhibit the transport of triglycerides and cholesterol across the intestine is a plausible explanation of their capacity to reduce LDL-C levels [5]. The consequences of the increased viscosity of the luminal contents manifest via the amplification of the thickness of the water layer and in the decrease of cholesterol uptake from the intestinal lumen [6].

The contribution of beta-glucans from oat and barley to the maintenance of normal blood cholesterol levels and their efficacy in the reduction of blood cholesterol levels at a dosage of 3 g per day was formally recognized by the European Food Safety Authority (EFSA) following specific applications and health claims that were authorized in the European Union (Commission Regulation (EU) 432/2012) [7]. However, the available clinical trials investigated the short-term lipid-lowering effect of beta-glucan administration on relatively small population samples and rarely involved European subjects [8,9]

In this context, we deemed interesting to evaluate the effect of a proprietary formulation of beta-glucans, at the dosage of 3 g/day on fasting plasma lipids and glucose, as well as its tolerability, in an Italian sample of subjects with mild hypercholesterolemia.

## 2. Materials and Methods

### 2.1. Study Design and Participants

The Beta-glucan Effects on Lipid profile, glycaemia and inTestinal health (BELT) Study was a medium-term, double-blind, placebo-controlled, cross-over randomized clinical trial, which enrolled a sample of Italian free-living subjects with moderate hypercholesterolemia recruited from the Lipid clinic of the S. Orsola-Malpighi University Hospital, Bologna, Italy.

Participants were required to be aged between 20 and 65 years, with moderately high levels of TC (TC ≥ 5.17 mmol/L and ≤ 6.2 mmol/L) and LDL-C (LDL-C ≥ 3.36 mmol/L and ≤ 4.91 mmol/L) and an estimated 10-year cardiovascular risk <10%, as per the country-specific risk charts from the CUORE project [10]. Exclusion criteria included having previously experienced any vascular event, suffering from type 1 or type 2 diabetes, massive hypertriglyceridemia (TG > 4.52 mmol/L), alcoholism, obesity (body mass index (BMI) > 30 kg/m^2^), liver failure, renal failure (estimated glomerular filtration rate (eGFR) < 0.5 mL/s), irritable bowel syndrome, inflammatory bowel diseases and food allergies, as well as having been treated in the previous 2 months with fiber based dietary supplements and/or probiotics and/or lipid-lowering drugs or any other drugs potentially able to affect the lipid metabolism.

**Figure 1:**
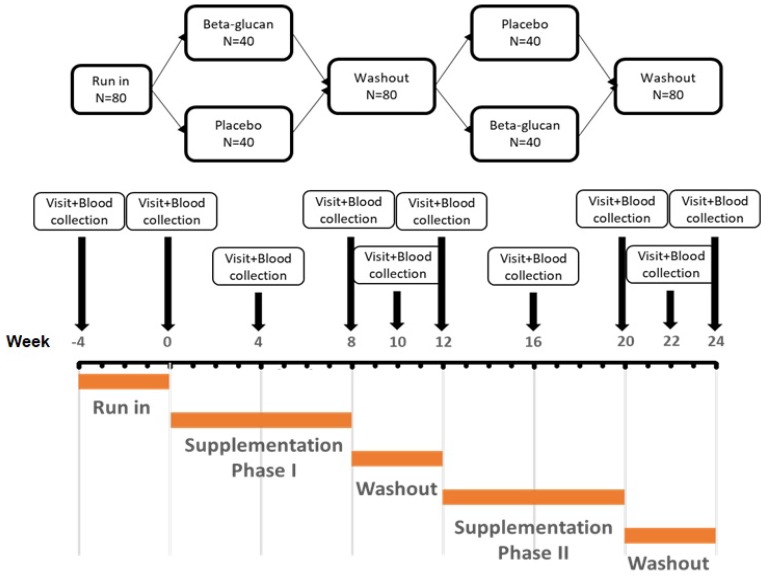
The flow-chart summarizes the study timeline.

Participants were adhering to a standardized diet for four weeks before being randomized to receive adequate supplies of either the beta-glucan supplement or placebo, in order to complete the one of two 2-month treatment sequences. The crossover to the second treatment was preceded by a 4-week wash-out period. Finally, participants were asked to return for follow-up visits two and four weeks after stopping supplementation. The study timeline is described in detail in Figure 1.

At enrollment, subjects were instructed to follow the general indications of a Mediterranean diet, avoiding an excessive intake of diary and red meat derived products.

Once every two visits, subjects were provided with a food diary to record their 3-day intake (two week-days and one week-end day), which they were requested to complete and return during their subsequent visit. In particular, the food diaries were collected and the diets analyzed at the beginning and at the end of each treatment phase.

The analysis of diet composition was performed using a dedicated software (MètaDieta^®^) based on a large food database that is frequently updated with values from the main official Italian databases (INRAN/IEO 2008 revision/ADI). Data were handled in compliance with the company procedure IOA87.

The study was conducted in accordance with the Good Clinical Practices and fully complied with the ethical guidelines of the Declaration of Helsinki. All subjects received an informational document describing the study and signed a consent form for study participation. The study was approved by the Bologna University Ethical Committee (Code: BELT_2016) and registered on www.clinicaltrial.gov (ID: NCT03313713).

### 2.2. Treatment

At visit at the beginning of supplementation the enrolled subjects were provided with boxes containing 28 white sachets of 15 g each; the same amount of product was provided after four weeks and at the beginning and after four weeks of the second supplementation phase. The sachets contained either 3 g oat beta-glucan (The Oatwell™ based Beta Heart^®^, Herbalife) or an oat-based isocaloric placebo without beta-glucan. The tested products had a similar macronutrient composition (Total energy: 50 kcal; Lipids: 1.4 g; Carbohydrates: 4 g; Proteins: 2 g) and were indistinguishable in color and taste. In particular, the Oatwell™ composition per 100 gr included 22 gr of beta-glucan soluble fiber characterized by very high molecular weight polysaccharides (>2000 kDa) and high viscosity (Table 1) [11].

Randomization was performed centrally, by computer-generated codes, and blocks were stratified by sex and age. The study staff and the investigators were blinded to the group assignment, as well as all the enrolled volunteers.

For the entire duration of the study, the subjects were instructed to take the dietary supplement regularly, dissolving the contents of one sachet in a glass of water every day in the morning.

All unused sachets were retrieved for inventory, and product compliance was assessed by counting the number of the sachets returned at the time of specific clinic visits [12]. The acceptability of the food supplements was assessed by a 10-point visual analogue score (VAS).

### 2.3. Assessments

#### 2.3.1. Clinical Data and Anthropometric Measurements

Subjects’ personal history was evaluated taking particular attention to cardiovascular and metabolic diseases, dietary and smoking habits assessment (both evaluated with validated semi-quantitative questionnaires) [13], physical activity, and pharmacological treatments.

Waist circumference (WC) was measured at the end of a normal expiration, in a horizontal plane at the midpoint between the inferior margin of the last rib and the superior iliac crest. Height and weight were measured to the nearest 0.1 cm and 0.1 kg, respectively, with subjects standing erect with eyes directed straight wearing light clothes and with bare feet. BMI was calculated as body weight in kilograms, divided by height squared in meters (kg/m^2^).

#### 2.3.2. Blood Pressure Measurements

Systolic and diastolic blood pressure measurements were performed in each subject, supine and at rest, using a validated oscillometric device, with a cuff of the appropriate size applied on the right upper arm. To improve detection accuracy, three blood pressure (BP) readings were sequentially obtained at 1-min intervals. The first one was then discarded, and the average between the second and the third was recorded [14].

#### 2.3.3. Laboratory Data

The biochemical analyses were carried out on venous blood, withdrawn after overnight fasting (at least 12 h). Plasma was obtained by addition of disodium ethylenediaminetetraacetate (Na_2_EDTA) (1 mg/mL) and blood centrifugation at 3000 RPM for 15 min at room temperature.

Trained personnel performed laboratory analyses according to standardized methods [15], immediately after centrifugation, to assess TC, HDL-C, TG, Apo-B, apolipoprotein A1 (Apo-A1), fasting plasma glucose (FPG), creatinine, estimated glomerular filtration rate (eGFR) and liver transaminases. LDL-C was obtained by the Friedewald formula. Non-HDL-C resulted from the difference between TC and HDL-C.

#### 2.3.4. Safety and Tolerability

Safety and tolerability were evaluated through continuous monitoring in order to detect any adverse event, clinical safety, laboratory findings, vital sign measurements, and physical examinations. A blinded, independent expert clinical event committee was appointed by the principal investigator in order to categorize the adverse events that could possibly be experienced during the trial as not related, unlikely related, possibly related, probably related, or definitely related to the study treatment.

Even if the fiber amount supplemented was low to modify the bowel function, an *ad-hoc* semi-quantitative questionnaire (Intestinal function assessment (Supplementary Material)) was administered at each visit, in order to collect information regarding possible changes in self-perceived intestinal well-being during the different phases of the study. The questionnaire evaluated the number of daily evacuations, the stool consistency and the personal perception of discomfort, swelling, stool expulsion ease and complete expulsion [16].

#### 2.3.5. Statistical Analyses

Sample size was calculated for the change in LDL-C. Considering a type I error of 0.05, a power of 0.80 and expecting a LDL-C reduction of 7–10% with beta-glucans and 0–3% with placebo, expecting a 20% dropout rate, we calculated to enroll 80 subjects.

Data were analyzed using intention to treat by means of the Statistical Package for Social Sciences (SPSS) version 22.0 (IBM Corporation, Armonk, NY, USA) for Windows.

A full descriptive analysis of the collected parameters was carried out as per protocol. Categorical variables were expressed as absolute number and percentage and compared with the Fisher corrected chi-square test or the Wilcoxon-Rank test, based on whether they were nominal or ordinal. Continuous variables were expressed as mean ± standard deviation (SD) or mean and standard error (SEM), and compared by analysis of variance (ANOVA) followed by post-hoc Tukey test or by Kruskal-Wallis non parametric analysis of variance followed by Dunn’s pairwise test, depending on their statistical distribution (if it was normal or not). To verify the basic assumption of cross-over design, the presence of a carryover effect was excluded.

The minimum level of statistical significance was set to *p* < 0.05 two-tailed. The Dixon’s Q test was always carried out to exclude the extreme values.

Efficacy analyses were performed considering the intention-to-treat (ITT) population, i.e., all subjects with at least one post-baseline control. A sensitivity analysis of the primary variable was also planned in the per-protocol population, i.e., all subjects without major protocol violations.

The primay efficacy outcome analysis was also repeated by gender, age group (20–40 vs. 40–65 years old) and weight group (body mass index < 27 kg/m^2^ vs. ≥ 27 kg/m^2^).

Safety results were reported in all subjects who had assumed at least one dose of one study supplement.

## 3. Results

A total of 95 volunteers were screened, and 83 subjects underwent randomization from April through September 2017.

Subjects’ characteristics at the screening visit are summarized in Table 2.

All enrolled subjects (men: 35, women: 48) successfully completed the trial according to the study design. The final distribution between men and women, when considering the treatment sequences, did not show any significant differences (*p* > 0.05). Furthermore, the study groups were well matched for all the considered variables at baseline.

No statistically significant changes were observed in the dietary habits of the enrolled subjects from randomization until the end of the study, nor in total energy and macronutrient intake (Table 3).

During the placebo phase, the subjects did not experience any statistically significant change in the evaluated parameters (Table 4 and Table S1 (Supplementary Materials)).

On the contrary, the active supplementation reduced mean LDL-C levels from baseline by 12.2% (95%CI: −15.4 to −3.8) after 4 weeks and by 15.1% (95%CI: −17.8 to −5.9) after 8 weeks (*p* < 0.01 for both comparison), which corresponded to an absolute decrease of 0.59 mmol/L (95%CI: −0.8 to −0.39) at the end of the intervention period (Table S2 (Supplementary Materials)). Repeating the analysis by gender and predefined age and body mass index groups, after 8 weeks we observed a slight but significantly higher LDL-lowering effect of beta-glucans effect in women than in men (women: 16.3% (95%CI: −17.8 to −6.7) vs. men: 14.9% (95%CI: −14.1 to −5.9); *p* = 0.04), in younger subjects (16.4% (95%CI: −17.5 to −8.3) vs. older 14.7% (95%CI: −17.1 to −5.2)) while no difference has been detected as regards body mass index classes (*p* > 0.05).

Considering the first-ranked secondary endpoints, the mean percentage change in TC levels between baseline and 4 weeks was a reduction of 6.5% (95%CI: −10.9 to −1.9) in the beta-glucan sequence, while non-HDL-C plasma concentrations decreased by 11.8% (95%CI: −14.6 to −4.5). Moreover, after 8 weeks of treatment, beta-glucan reduced TC by 8.9% (95%CI: −12.6 to −2.3), corresponding to an absolute decrease of 0.52 mmol/L (95%CI: −0.72 to −0.32), and non-HDL-C levels were reduced by 12.1% (95%CI: −15.6 to −5.3), which corresponded to 0.53 mmol/L (95%CI: −0.73 to −0.33) (Table S2 (Supplementary Materials)).

Descriptions and results of exploratory analyses of the other secondary endpoints, the effect on which were considered non-significant, are provided in Table 5.

After treatment discontinuation, both TC and LDL-C rapidly reversed to basal values. Lipid plasma levels assessed after 2 weeks of washout were comparable with those measured at baseline and not significantly different from those observed at the end of the wash-out period (Table 5).

The compliance with the treatment was almost complete (89%) during both treatment periods.

The tolerability profile of the beta-glucan supplement used was rated as acceptable by most subjects. During supplementation with beta-glucan, three subjects experienced moderate abdominal discomfort (reported as reversible abdominal cramps and diarrhoea) and one subjects experienced dysphagia. No one experienced adverse events regarding laboratory parameters during the trial. No volunteer discontinued the trial because of adverse events that occurred during the treatment and no effects reported in conjunction with use of the placebo.

In particular, the tested products did not exert any significant unvafourable effect on the self-perceived intestinal well-being. The results of the second-ranked secondary endpoint are summarized in Table 6.

## 4. Discussion

Beta-glucans (1-3,1-4 beta-D-glucans) are polysaccharides naturally occurring in the cell wall of grains and cereals, especially barley and oats [17]. In our double-blind, placebo-controlled, cross-over randomized clinical trial, 3 g/day of oat beta-glucan were shown to safely reduce LDL-C, TC and non-HDL-C in a large sample of adults characterized by mild hypercholesterolemia and with a low cardiovascular risk profile. The trial failed to detect any significant change in FPG. On the other hand, no significant adverse effects have been assessed on the self-perceived intestinal wellbeing and of both the beta-glucan supplement and the placebo were considered accettable.

The observed effect on TC and LDL-C are larger (0.53 and 0.59 mml/L, on average, corresponding to 15.1% and 8.9% of baseline values respectively) than expected, based on the most recent meta-analysis and EFSA opinion, which estimate respectively a mean change in LDL-C of 0.3 mmol/L and 0.21 mmol/L (about 7–10% of baseline concentratios) [7,18]. The reasons of such better performance are not easily explained, but it should be considered the possibility that the tested formulation, to be dissolved in fluids before consumption, might have specific pharmaceutic properties able to enhance the efficacy of the fibers in binding cholesterol and/or its metabolites (i.e., bile salts) and/or dietary fat. However, the supply of oat beta-glucan in the form of beverages (where nutrients are in contact with free water) has been found to have, in general, a more regular effect on cholesterol reduction compared to more complex matrices [19]. Moreover, the beta-glucan used for the BELT study consists of very high molecular weight beta-glucan, which has been proved to be more effective in cholesterol lowering then that with medium and low molecular weight [11,20].

Furthermore, the observed effect might also be due to the characteristics of the considered population sample. In this regard, a reliable meta-regression analysis recently demonstrated a significant inverse association between baseline LDL-C levels and the extent of LDL-C reduction after supplementation with oat beta-glucan [2]. These results are of particular interest, also considering that the observed effect is larger than the one expected after the administration of most available lipid-lowering nutraceuticals [21,22].

Similarly, the lack of any significant effect on FPG in the trial might be due to the characteristics of the enrolled subjects, being all euglycemic. Actually, a recent meta-analysis found that oat beta-glucan intake was more effective in people with type 2 diabetes [23].

The lack of changes in metabolic parameters during supplementation with placebo may be attributed to both the enrollment in the setting of a lipid clinic (and consequently, presumably, of subject *a priori* more attentive to a healthy lifestyle) and to the run-in stabilization diet period preceding the treatment phase, during which possible dietary mistakes were corrected before the start of the trial. Thus, the results obtained with beta-glucan supplementation are more representative of what would be observed in a setting of clinical practice.

At the same time, the lack of effects of beta-glucans on intestinal function parameters in our study should be related to the Mediterranean diet pattern of the enrolled subjects and the exclusion from the enrolment of subjects affected by irritable bowel syndrome, inflammatory bowel diseases and food allergies, but also because of the low amount of supplemented fibers.

The observed results are largely supported by the mechanisms of action by wich beta-glucans can improve cholesterolemia. The LDL-C lowering effect of beta-glucans has mainly related to the their ability to act as dietary fibers, consequently entrapping bile acid micelles, impairing their ability to interact with luminal membrane transporters on the intestinal epitelium, thereby increasing the fecal cholesterol output. [24] The following decrease in bile acid level up-regulates the 7-alpha-hydroxylase expression in the liver, thus further contributing to LDL-C decrease. [11] This seems to be more evident with high molecular weight and high viscosity of the fibers [25], as the one we tested in our trial. More recent literature suggesta that a part of the LDL-C reducing effect of beta-glucans could be mediated by modulation of microbiota [26]. A part of the beta-glucans effects (i.e., the antinflammatory and immunomodulatory ones) seems to be dependent by the contact of beta-glucans with dectin-1 [27], but this could be less relevant for th metabolic activities.

The BELT Study has some relevant limitations. For instance, the observational period was not long enough to assess any adaptation phenomena possibly occurring by changes in the gut microbiota composition. For this reason, further longer-term clinical studies are needed to confirm if the dietary intake of beta-glucan enriched foods is able to maintain the observed positive metabolic effect over the time.

Secondly, we did not evaluate any marker of intestinal absorption during the study, since the aim of this study was a clinical and not a pharmacological evaluation of the effects of supplementation with beta-glucan-enriched dietary supplement in moderately hypercholesterolemic subjects.

However, the BELT Study is the first placebo-controlled clinical trial testing the effect of oat beta-glucan supplementation on a large sample of a South European population strictly adherent to the Mediterranean diet. This is of particular interest because the high fiber content of the Mediterranean diet might have theoretically reduced the effect of beta-glucan supplementation on serum lipids [28], while this actually did not occur. Moreover, the observation that both total and LDL-C concentrations tend to return to basal values after weeks of wash-out, highlights the importance of a regular and constant supplementation with beta-glucan to achieve clinically relevant results in the long term. Finally, the profile of tolerability of the product might suggest that compliance to long term treatment with beta-glucan formulation might be good.

## 5. Conclusions

In conclusion, the BELT study confirms the medium-term efficacy of supplementation with 3 g/day of beta-glucan in reducing LDL-C, TC and non-HDL-C in mild hypercholesterolemic subjects, even in the context of a Mediterranean setting.

## Figures and Tables

**Table 1:** Nutritional composition per 100 g of OatWell^®^ 22.

Total dietary fiber	44 g
- Glucan soluble fiber	22 g
Carbohydrate	22 g
Protein	20 g
Total lipids	5 g
- Saturated FA	1 g
- Polyunsaturated FA	2 g
- Monounsaturated FA	2 g
Water	5 g
Minerals	
- Sodium (Na)	3 mg
- Magnesium (Mg)	250 mg
- Calcium (Ca)	120 mg
- Potassium (K)	700 mg
- Iron (Fe)	9 mg
- Zinc (Zn)	6 mg

**Table 2:** Pre-diet standardization parameters, expressed as mean ± SD.

Parameters	Pre-Diet Standardization (*N* = 83)
Age (years)	52.3 ± 4.4
Weight (kg)	74.5 ± 17.4
Waist (cm)	91.3 ± 14.6
Systolic blood pressure (mmHg)	128.3 ± 15.3
Diastolic blood pressure (mmHg)	81 ± 9.6
Total cholesterol (mmol/L)	5.75 ± 0.49
Triglycerides (mmol/L)	1.51 ± 0.80
HDL-cholesterol (mmol/L)	1.28 ± 0.29
Non-HDL-cholesterol (mmol/L)	4.48 ± 0.49
LDL-cholesterol (mmol/L)	3.78 ± 0.42
VLDL-cholesterol (mmol/L)	0.33 (0.32)
Apolipoprotein-A1 (mg/dL)	149.3 ± 22.9
Apolipoprotein -B (mg/dL)	100.8 ± 18.5
Fasting plasma glucose (mmol/L)	4.97 ± 0.73
Aspartate aminotransferase (μkat/L)	0.38 ± 0.12
Alanine aminotransferase (μkat/L)	0.39 ± 0.24

HDL = High-Density Lipoprotein; LDL = Low-Density Lipoprotein; VLDL = Very-Low-Density Lipoprotein.

**Table 3:** Diet composition and total fiber intake (g/day) at the enrollment. Values are reported as mean ± SD or mean and variation range.

Parameters	Diet Composition
Total Energy (kcal/day)	1388.7 ± 245.7
Alcohol (% of total energy)	4.4 (0.1–10.4)
Lipids (% of total energy)	32.9 ± 4.9
Saturated Fatty Acids (% of total energy)	7.9 ± 2.4
Monounsaturated Fatty Acids (% of total energy)	12.4 ± 3.1
Poliunsaturated Fatty Acids (% of total energy)	4.4 ± 1.1
Proteins (% of total energy)	18.3 ± 5
Animal Proteins (% of total energy)	10.2 ± 6
Vegetal Proteins (% of total energy)	5.1 ± 2.1
Carbohydrates (% of total energy)	50.4 ± 5.9
Soluble Charbohydrates (% of total energy)	15.9 ± 3.7
Starch (% of total energy)	25.3 ± 4.5
Total dietary fibers (% of total energy)	2.2 ± 0.9
Cholesterol (mg/day)	148.4 ± 22.3

**Table 4:** Anthropometric, hemodynamic, and blood chemistry parameters from the baseline/end of wash-out to the end of the placebo sequence, expressed as mean ± SD.

Parameters	Baseline	Treatment Period		Wash-Out Period	
		4 Weeks	8 Weeks	2 Weeks	4 Weeks
Weight (kg)	73.8 ± 17.2	73.8 ± 17.1	73.7 ± 17.1	73.8 ± 17.9	74 ± 17.2
Waist (cm)	90.9 ± 14.4	90.8 ± 14.3	90.6 ± 14	90.6 ± 14.9	90.7 ± 14.1
Systolic blood pressure (mmHg)	120.9 ± 15.8	120.9 ± 15.2	120.3 ± 15.8	120.2 ± 16.7	121.1 ± 15.1
Diastolic blood pressure (mmHg)	78.5 ± 8.6	78.8 ± 8.6	79.2 ± 9.9	78.1 ± 8.4	78 ± 9.1
Heart rate (bpm)	71.9 ± 10.6	66.3 ± 4.7	70.78 ± 12.9	72.6 ± 10.2	70.2 ± 9.6
Total cholesterol (mmol/L)	5.75 ± 0.77	5.84 ± 0.73	5.75 ± 0.75	5.79 ± 0.81	5.85 ± 0.87
Triglycerides (mmol/L)	1.48 ± 0.70	1.46 ± 0.88	1.4 ± 0.78	1.59 ± 0.81	1.5 ± 0.76
HDL-cholesterol (mmol/L)	1.31 ± 0.29	1.33 ± 0.29	1.31 ± 0.3	1.26 ± 0.3	1.34 ± 0.28
LDL-cholesterol (mmol/L)	3.76 ± 0.75	3.84 ± 0.65	3.8 ± 0.65	3.81 ± 0.8	3.83 ± 0.80
Non HDL-cholesterol (mmol/L)	4.44 ± 0.71	4.51 ± 0.73	4.44 ± 0.72	4.53 ± 0.75	4.51 ± 0.83
Apolipoprotein A1 (mg/dL)	157.3 ± 32.5	151.2 ± 25.7	151.6 ± 26. 5	148.4 ± 25.7	161 ± 29.2
Apolipoprotein B (mg/dL)	101.4 ± 22.7	101.6 ± 15.3	99.8 ± 17.6	102.9 ± 14.4	100.3 ± 17.1
VLDL-cholesterol (mmol/L)	0.68 ± 0.32	0.71 ± 0.65	0.68 ± 0.62	0.73 ± 0.37	0.69 ± 0.35
Fasting plasma glucose (mmol/L)	5.01 ± 0.56	5.06 ± 0.50	5.02 ± 0.49	4.99 ± 0.51	5.09 ± 0.59
Aspartate aminotransferase (μkat/L)	0.36 ± 0.09	0.37 ± 0.11	0.37 ± 0.12	0.36 ± 0.10	0.35 ± 0.08
Alanine aminotransferase (μkat/L)	0.36 ± 0.17	0.4 ± 0.27	0.44 ± 0.37	0.4 ± 0.18	0.41 ± 0.20

HDL = High-density lipoprotein; LDL = Low-density lipoprotein; VLDL = Very-low-density lipoprotein.

**Table 5:** Changes in anthropometric, hemodynamic, and blood chemistry parameters from the baseline/end of washout to the end of the supplementation with beta-glucan, expressed as mean ± SD.

Parameters	Baseline	Treatment Period		Wash-Out Period	
		4 Weeks	8 Weeks	2 Weeks	4 Weeks
Weight (kg)	73.9 ± 17.6	73.8 ± 17.7	73.4 ± 17.6	73.9 ± 14.6	73.6 ± 17.7
Waist (cm)	90.3 ± 14.2	90.2 ± 14.0	89.9 ± 13.7	88.5 ± 13.7	90.3 ± 13.8
Systolic blood pressure (mmHg)	124.3 ± 16.0	121.1 ± 14.1	119.5 ± 15.2	122.2 ± 16.5	120.7 ± 14.2
Diastolic blood pressure (mmHg)	80.4 ± 9.6	78.6 ± 8.8	79.4 ± 9	80.4 ± 8.9	78.4 ± 9.4
Heart rate (bpm)	72.1 ± 11.5	68 ± 1.4	72.3 ± 12.9	72.1 ± 11.8	72 ± 8.2
Total cholesterol (mmol/L)	5.77 ± 0.68	5.38 ± 0.58 *^,^°	5.24 ± 0.57 *^,^°	5.76 ± 0.71	5.81 ± 0.76
Triglycerides (mmol/L)	1.48 ± 0.81	1.6 ± 0.89	1.62 ± 1.10	1.46 ± 0.80	1.52 ± 0.72
HDL-cholesterol (mmol/L)	1.29 ± 0.33	1.32 ± 0.29	1.3 ± 0.29	1.28 ± 0.32	1.33 ± 0.29
LDL-cholesterol (mmol/L)	3.8 ± 0.64	3.33 ± 0.60 *^,^^§^	3.21 ± 0.65 *^,§^	3.82 ± 0.64	3.78 ± 0.74
Non HDL-cholesterol (mmol/L)	4.48 ± 0.67	4.07 ± 0.60 *^,§^	3.95 ± 0.62 *^,§^	4.49 ± 0.66	4.48 ± 0.73
Apolipoprotein A1 (mg/dL)	151.7 ± 27.2	149.1 ± 27.5	147.33 ± 26.9	150.1 ± 25.7	166.8 ± 27.6
Apolipoprotein B (mg/dL)	99.2 ± 16.7	101.5 ± 16	100.1 ± 15.4	96.9 ± 14.6	102.8 ± 14.4
VLDL-cholesterol (mmol/L)	0.66 ± 0.32	0.73 ± 0.41	0.81 ± 0.83	0.67 ± 0.36	0.7 ± 0.33
Fasting plasma glucose (mmol/L)	4.78 ± 0.55	5.05 ± 0.48	5.02 ± 0.52	4.97 ± 0.51	5.07 ± 0.52
Aspartate aminotransferase (μkat/L)	0.38 ± 0.10	0.38 ± 0.11	0.37 ± 0.1	0.37 ± 0.10	0.36 ± 0.10
Alanine aminotransferase (μkat/L)	0.37 ± 0.18	0.42 ± 0.21	0.42 ± 0.22	0.36 ± 0.18	0.42 ± 0.20

* *p* < 0.05 versus baseline; ° *p* < 0.05 versus placebo; ^§^ *p* < 0.01 versus placebo. HDL = High-Density Lipoprotein; LDL = Low-Density Lipoprotein; VLDL = Very-Low-Density Lipoprotein.

**Table 6:** Pre-versus post-treatment effects on intestinal function of the tested products.

Parameters	Placebo		Beta-Glucan	
	Mean	*p*	Mean	*p*
Number of defecations per week	7	0.264	7	0.730
Stool consistency	3	0.482	3	0.438
Easiness of stool expulsion	4	0.610	3	0.699
Perception of total expulsion	3	0.691	4	0.583
Abdominal discomfort intensity	2	0.744	2	0.923
Abdominal swelling perception	3	0.749	3	0.760

## Supplementary Materials

The following are available online at https://www.mdpi.com/2072-6643/12/3/686/s1, Table S1: Changes in the collected parameters during supplementation with placebo in the overall study population, Table S2: Changes in the collected parameters during supplementation with beta-glucan in the overall study population.
