# Supplementary material for: A Randomized Placebo-Controlled Clinical Trial to Evaluate the Medium-Term Effects of Oat Fibers on Human Health: The Beta-Glucan Effects on Lipid Profile, Glycemia and inTestinal Health (BELT) Study
Source: Nutrients. 2020 Mar 3;12(3):686. doi: 10.3390/nu12030686 (PMC7146517; doi:10.3390/nu12030686)
Supplement: Supplementary file 1 [file nutrients-12-00686-s001.pdf]

Intestinal function assessment

Code _ _ _ _ _ _

Name

____

Surname

____

Date _ _ / _ _ / 20 _ _

Visit n. _ _

Number of defecations per week _ _

Stool consistency

Low (liquid)		Regular		High (hard)
1	2	3	4	5

Easiness of stool expulsion

Low				High
1	2	3	4	5

Perception of total expulsion

Low				High
1	2	3	4	5

Abdominal discomfort intensity

Low				High
1	2	3	4	5

Abdominal swelling perception

Low				High
1	2	3	4	5

Table S1. Changes in the collected parameters during supplementation with placebo in the overall study population.

Parameters	Time	Mean	SD	SEM	*p* (2-Tails)	Post-Treatment Mean Difference	95% Confidence Interval	
							Lower	Upper
Weight (Kg)	Baseline	73.8	17.2	1.9	0.947	−0.2	−5.4	5.1
	End of treatment	73.7	17.1	1.9				
Waist (cm)	Baseline	90.9	14.4	1.6	0.868	−0.4	−4.7	4
	End of treatment	90.6	14	1.5				
Systolic blood pressure (mmHg)	Baseline	120.9	15.8	1.7	0.802	−0.6	−5.4	4.2
	End of treatment	120.3	15.8	1.7				
Diastolic blood pressure (mmHg)	Baseline	78.5	8.6	0.9	0.615	0.7	−2.1	3.6
	End of treatment	79.2	9.9	1.1				
Heart rate (bpm)	Baseline	71.9	10.6	2.1	0.692	−1.1	−6.8	4.6
	End of treatment	70.8	12.9	1.7				
Total cholesterol (mmol/L)	Baseline	5.75	0.77	0.08	0.987	0	−0.24	0.23
	End of treatment	5.75	0.75	0.08				
Triglycerides (mmol/L)	Baseline	1.48	0.7	0.08	0.493	−0.08	−0.31	0.15
	End of treatment	1.4	0.78	0.09				
HDL-cholesterol (mmol/L)	Baseline	1.31	0.29	0.03	0.914	0	−0.1	0.09
	End of treatment	1.31	0.3	0.03				
LDL-cholesterol (mmol/L)	Baseline	3.76	0.75	0.08	0.718	0.04	−0.18	0.25
	End of treatment	3.8	0.65	0.07				
Non HDL-cholesterol (mmol/L)	Baseline	4.44	0.71	0.08	0.978	0	−0.22	0.22
	End of treatment	4.44	0.72	0.08				
Apolipoprotein-A1 (mg/dl)	Baseline	157.3	32. 5	3. 6	0.217	−5.7	−14.8	3.4
	End of treatment	151.6	26. 4	2.9				
Apolipoprotein-B (mg/dl)	Baseline	101.4	22.7	2.5	0.623	−1.6	−7.8	4.7
	End of treatment	99.8	17.6	1.9				
VLDL-cholesterol (mmol/L)	Baseline	0.68	0.32	0.04	0.977	0	−0.15	0.15
	End of treatment	0.68	0.62	0.07				
Fasting plasma glucose (mmol/L)	Baseline	5.01	0.56	0.06	0.922	0.01	−0.15	0.17
	End of treatment	5.02	0.49	0.05				
Aspartate aminotransferase (μkat/L)	Baseline	0.36	0.09	0.01	0.583	0.01	−0.02	0.04
	End of treatment	0.37	0.12	0.01				
Alanine aminotransferase (μkat/L)	Baseline	0.36	0.17	0.02	0.095	0.07	−0.01	0.16
	End of treatment	0.44	0.37	0.04				

HDL-C = High-density lipoprotein; LDL-C = Low-density lipoprotein; SD = Standard deviation; SEM = Standard error of the mean; VLDL = Very-low-density lipoprotein.

Table S2. Changes in the collected parameters during supplementation with beta-glucan in the overall study population.

Parameters	Time	Mean	SD	SEM	*P* (2-Tails)	Post-Treatment Mean Difference	95% Confidence Interval	
							Lower	Upper
Weight (Kg)	Baseline	74	17.6	2	0.848	−0.5	−6.1	5
	End of treatment	73.4	17.6	2				
Waist (cm)	Baseline	90.3	14.2	1.6	0.856	−0.4	−4.8	4
	End of treatment	89.9	13.7	1.5				
Systolic blood pressure (mmHg)	Baseline	124.3	16	1.8	0.053	−4.8	−9.7	0.1
	End of treatment	119.5	15.2	1.7				
	Baseline	80.4	9.6	1.1	0.492	−1	−3.9	1.9

Diastolic blood pressure (mmHg)	End of treatment	79.4	9	1				
Heart rate (bpm)	Baseline	72.1	11.5	1.7	0.938	0.2	-4.6	4.9
	End of treatment	72.3	12.9	1.7				
Total cholesterol (mmol/L)	Baseline	5.77	0.68	0.08	<0.001	−0.52	−0.72	−0.32
	End of treatment	5.24	0.57	0.06				
Triglycerides (mmol/L)	Baseline	1.48	0.81	0.09	0.385	0.13	−0.17	0.44
	End of treatment	1.62	1.1	0.12				
HDL-cholesterol (mmol/L)	Baseline	1.29	0.33	0.04	0.822	0.01	−0.09	0.11
	End of treatment	1.3	0.29	0.03				
LDL-cholesterol (mmol/L)	Baseline	3.8	0.64	0.07	<0.001	−0.59	−0.8	−0.39
	End of treatment	3.21	0.65	0.07				
Non HDL-cholesterol (mmol/L)	Baseline	4.48	0.67	0.07	<0.001	−0.53	−0.73	−0.33
	End of treatment	3.95	0.62	0.07				
Apolipoprotein-A1 (mg/dl)	Baseline	151.7	27.2	3	0.314	−4.3	−12.8	4.1
	End of treatment	147.3	26.9	3				
Apolipoprotein-B (mg/dl)	Baseline	99.2	16.7	1.9	0.714	0.9	−4.1	6
	End of treatment	100.1	15.4	1.7				
VLDL-cholesterol (mmol/L)	Baseline	0.66	0.32	0.04	0.144	0.15	-0.05	0.35
	End of treatment	0.81	0.83	0.09				
Fasting plasma glucose (mmol/L)	Baseline	4.78	0.55	0.06	0.816	0.02	−0.15	0.19
	End of treatment	5.02	0.52	0.06				
Aspartate aminotransferase (µkat/L)	Baseline	0.38	0.1	0.01	0.485	−0.01	−0.04	0.02
	End of treatment	0.37	0.1	0.01				
Alanine aminotransferase (µkat/L)	Baseline	0.37	0.18	0.02	0.123	0.05	−0.01	0.11
	End of treatment	0.42	0.22	0.02				

HDL-C = High-density lipoprotein; LDL-C = Low-density lipoprotein; SD = Standard deviation; SEM = Standard error of the mean; VLDL = Very-low-density lipoprotein.
